# Termiticidal Effects and Morpho-Histological Alterations in the Subterranean Termite (*Odontotermes formosanus*) Induced by Biosynthesized Zinc Oxide, Titanium Dioxide, and Chitosan Nanoparticles

**DOI:** 10.3390/nano14110927

**Published:** 2024-05-24

**Authors:** Raghda Nasser, Ezzeldin Ibrahim, Hatem Fouad, Farhan Ahmad, Wuhan Li, Qihuan Zhou, Ting Yu, Nooney Chidwala, Jianchu Mo

**Affiliations:** 1 Ministry of Agriculture Key Laboratory of Molecular Biology of Crop Pathogens and Insect Pests, Key Laboratory of Biology of Crop Pathogens and Insects of Zhejiang Province, Institute of Insect Sciences, College of Agricultural and Biotechnology, Zhejiang University, Hangzhou 310058, China; raghdanasser@mu.edu.eg (R.N.); 22116223@zju.edu.cn (W.L.); 11916108@zju.edu.cn (Q.Z.); yting@zju.edu.cn (T.Y.); nchidwala@outlook.com (N.C.); 2 Zoology and Entomology Department, Faculty of Science, Minia University, El-Minia 61519, Egypt; 3 State Key Laboratory of Rice Biology and Breeding, Ministry of Agriculture Key Laboratory of Molecular Biology of Crop Pathogens and Insects, Key Laboratory of Biology of Crop Pathogens and Insects of Zhejiang Province, Institute of Biotechnology, Zhejiang University, Hangzhou 310058, China; ezzelbehery8818@yahoo.com; 4 Department of Vegetable Diseases Research, Plant Pathology Research Institute, Agriculture Research Centre, Giza 12916, Egypt; 5 Department of Field Crop Pests, Plant Protection Research Institute, Agricultural Research Centre, Cairo 12622, Egypt; dr_hatem@zju.edu.cn; 6 Entomology Section, Central Cotton Research Institute, Multan P.O. Box 66000, Pakistan; farhanmhanif7@gmail.com

**Keywords:** ZnONPs, TiO_2_NPs, CsNPs, *Odontotermes formosanus*, *Scedosporium apiospermum*

## Abstract

Recently, nanoparticles have been widely used in agricultural pest control as a secure substitute for pesticides. However, the effect of nanoparticles on controlling the subterranean termite *Odontotermes formosanus* (*O. formosanus*) has not been studied yet. Consequently, this study aimed to evaluate the effectiveness of some nanomaterials in controlling *O. formosanus*. The results showed that zinc oxide nanoparticles (ZnONPs), titanium dioxide nanoparticles (TiO_2_NPs), and chitosan nanoparticles (CsNPs) biosynthesized using the culture filtrate of *Scedosporium apiospermum* (*S. apiospermum*) had an effective role in controlling *O. formosanus*. Moreover, the mortality rate of *O. formosanus* after 48 h of treatment with ZnONPs, TiO_2_NPs, and CsNPs at a 1000 µg/mL concentration was 100%, 100%, and 97.67%, respectively. Furthermore, using ZnONPs, TiO_2_NPs, and CsNPs on *O. formosanus* resulted in morpho-histological variations in the normal structure, leading to its death. X-ray diffraction, UV-vis spectroscopy, Fourier transform infrared spectroscopy, scanning electron microscopy, dynamic light scattering, energy dispersive spectroscopy, and the Zeta potential were used to characterize the biosynthesis of ZnONPs, TiO_2_NPs, and CsNPs with strong activity against *O. formosanus* termites. Overall, the results of this investigation suggest that biosynthesized ZnONPs, TiO_2_NPs, and CsNPs have enormous potential for use as innovative, ecologically safe pesticides for *O. formosanus* control.

## 1. Introduction

Termites pose a serious threat to plant and building survival, and they are a major pest in forestry and agriculture worldwide [1]. Statistics show that more than 2800 termite species have been identified worldwide, 185 of which are regarded as harmful pests [2], especially termites that belong to the families Hodotermitidae, Kalotermitidae, Rhinotermitidae, and Termitidae. *Odontotermes formosanus* Shiraki, a subterranean termite, is one of the most destructive species in the world [3]. Besides the great damage caused by *O. formosanus* workers consuming a variety of plants, shrubs, and weeds in their diet, they also causes severe damage to facilities, buildings, and dams, resulting in a loss of billions of dollars [4]. Previous studies have estimated that the annual expenditure on termite control has reached 22 billion dollars around the globe, which is increasing by 50% each year [5]. In the USA alone, termite control costs 175 million dollars annually [6]. Undoubtedly, one of the most popular and successful approaches to termite control is the employment of chemical-based insecticides [7]. However, overuse of pesticides has resulted in many issues, including biological resistance, ecological imbalances, and adverse effects on the environment [8]. 

Over the last two decades, nanotechnology has been one of the most extensive and vibrant research areas in science and technology [9]. Metal oxide and natural polymer nanoparticles have additional benefits, such as low harmful effects and excellent stability, chemical properties, and toxicity compared to other metal nanoparticles [10]. These advantages have prioritized their inclusion in various applications in biomedicine and biotechnology [11]. Zinc oxide (ZnO-NPs) and titanium dioxide (TiO_2_-NPs) are two of the metal oxide nanoparticles that have unique chemical, catalytic, and optical properties that make them widely used in several fields, such as skin care products, electronic products, solar energy cells, anti-bacterial coatings, and anti-pollution, without any detrimental effects [12,13]. Moreover, chitosan nanoparticles (CsNPs), a biopolymer derived from chitin, are remarkably flexible and offer excellent chemical properties [14]. In agriculture, these nanomaterials have been used to suppress many plant pathogens and pests, in addition to their great role in improving plant health [15]. However, their use in termite control to reduce termites’ harmful effects has not been sufficiently studied yet.

Nanomaterials can be fabricated by various methods, including physical, chemical, and biological methods. However, the physical and chemical processes are energy-intensive, time-consuming, and release toxic substances into the environment [16]. Conversely, the biological method is less complicated, quicker, less expensive, and more environmentally friendly than the chemical and physical methods [17,18,19]. Therefore, researchers concentrated on the use of various substances in biosynthesis, as plant extracts from the fruits, stems, flowers, leaves, and roots of some plants, as well as the use of metabolites of some microbial species, such as fungi, algae, yeast, bacteria, and phages, as safe alternatives to the environment [20,21]. Fungi are among the most important biological microbial agents used in the biosynthesis of nanoparticles because they can produce many metabolites, the most important of which are enzymes and proteins, which are used as strong reducing agents to convert elements into their nanoforms [22]. Additionally, because of their high biomass output, ease of handling, scalability, high efficacy for metal tolerance, and economic viability, fungi are prioritized for use as templates in the fabrication of different nanoparticles [23]. For example, many nanomaterials, such as FeNPs, AuNPs, AgNPs, CuNPs, ZnONPs, TiO_2_NPs, and CuONPs, as well as natural nanopolymers such as nanocellulose, nanogelatin, nanodextran, and nanochitosan have been produced using some fungal filters [20,21,24]. A filamentous fungus called *Scedosporium apiospermum* had previously been believed to be the asexual version of *Pseudoallescheria boydii*. This fungus, which is eutrophic, is typically found in soil. Polluted or manure-enriched environments, such as garden and agricultural soil, encourage the development of it [25,26]. However, the use of this fungus as a biological agent to synthesize nanomaterials to mitigate damage caused by termites has not been reported. The purpose of this investigation was to evaluate the effectiveness of ZnONPs, TiO_2_NPs, and CsNPs biosynthesized using the culture filtrate of *Scedosporium apiospermum* against *O. formosanus* as environmentally friendly pesticides.

## 2. Materials and Methods

### 2.1. Materials

The following chemicals were bought from Sigma Aldrich Chemical Corporation in China: zinc oxide (ZnO), titanium dioxide (TiO_2_), chitosan (Cs) from shrimp shells (75% deacetylated), sodium tripolyphosphate (TPP), sodium hydroxide (NaOH), and acetic acid (CH_3_COOH). The analytical chemicals and reagents were obtained from nearby enterprises. The total protein and enzyme quantification kits were acquired from Beyotime Biotechnology, China. For the preparation of all the solutions, ultrapure water with a resistivity of at least 18.2 MΩ·cm was procured from YJD UPWS Ultra-Pure Water System, Technology Co., Ltd., Shenzhen, China.

### 2.2. Gathering and Raising Termite Colonies

In a wooded area in Hangzhou city, Zhejiang province, China, three colonies of *O. formosanus* (5–8 cm in height, 4–10 cm in diameter), each of them with a drone and queen, were gathered. Within ten hours after digging, the colonies and combs were encased in a film of plastic and brought to the laboratory. Each colony was arranged independently in plastic chambers filled with clay soil that was taken from the colony collection site. The rearing systems were kept at 28 ± 1 °C and greater than 90% relative humidity in total darkness. Once the colonies had stabilized in the lab, they were supplied with sweet osmanthus wood, *Osmanthus fragrans*.

### 2.3. Isolation of Rhizosphere Fungi

Between December 2022 and January 2023, nine soil samples were collected from several locations in Hangzhou, China. Each sample contained soil attached to the roots of *O. fragrans*. The samples were placed in a plastic bag, shaken carefully to separate the soil from the roots, and immediately transferred to a cooler until reaching the laboratory, where they were stored at 4 °C. The rhizosphere fungi were separated following the technique of Ru and Di [27], with slight changes. Soil samples were diluted to different concentrations by dissolving 1 g of soil per sample well in 9 mL of saline solution (0.9% NaCl) until a dilution of 10^−4^. The supernatants were then collected, and 250 µL of each dilution was decanted into potato dextrose agar (PDA) medium and incubated at 28 °C. After five days of incubation, the colonies were purified by transferring the single colonies to fresh PDA plates for nanoparticle biosynthesis.

### 2.4. Identification of Rhizosphere Fungi

Fungi isolated from *O. fragrans* were identified following the technique of Stracquadanio et al. [28], with some minor adjustments. The isolated fungi were cultivated on PDA plates for five days, mycelia were collected, and DNA purification and extraction were carried out using the Genomic Fungal DNA Isolation Kit per the guidelines given in the experiment’s protocol. The PCR product was sequenced by the SUNYA Biotechnology sequencing department (Hangzhou, China). Sequences were aligned by the NCBI blast tool on 13 March 2023 (http://www.ncbi.nlm.nih.gov) [29]. The Mega 6 program’s neighbor-joining approach was used to construct a phylogenetic tree, and the sequence was then added to the NCBI database.

### 2.5. Preparation of the Culture Filtrate 

The culture filtrate (CF) of fungal isolates that were isolated from the *O. fragrans* rhizosphere was prepared using the strategy of Stracquadanio et al. [28], albeit with a few little adjustments. In a nutshell, three plugs (diameter 5 mm) from the actively growing edges of the PDA culture for each isolate were inoculated individually into 100 mL flasks with 50 mL of potato dextrose broth (PDB). For 8 days at 28 °C, the flasks were shaken at 100 rpm on a rotary shaker. To obtain pure CF, the cultures were filtered under a vacuum through filter paper and twice through a 0.22 µm filter.

### 2.6. Biosynthesis of Nanoparticles

#### 2.6.1. Biosynthesis of ZnONPs and TiO_2_NPs 

The mycosynthesis of ZnONPs and TiO_2_NPs was performed using the techniques of Mishra et al. [30], with minor adjustments. Briefly, 100 mL of a solution of 0.1 M ZnO and 0.1 M TiO_2_ was mixed separately with 100 mL of CF from *S. apiospermum*. While stirring on a magnetic stirrer for 30 min, the ZnO mixture received 100 mL of 0.4 M NaOH, and then the combination was heated in a bath of water for 30 min at 37 °C, while the TiO_2_ mixture was stirred for 4 h on a magnetic stirrer at 1200 rpm at 37 °C. The pellets were gathered by centrifugation at 4500 rpm for 25 min, washed with ddH_2_O, and dried at 82 °C for 24 h.

#### 2.6.2. Biosynthesis of CsNPs

The mycosynthesis of CsNPs was performed using the approach of Dipali et al. [31], with a few adjustments. Briefly, TPP solution (0.8%) was added dropwise to 1.5% chitosan (1% acetic acid) under magnetic stirring. Afterward, 100 mL of CF of *S. apiospermum* was added dropwise to 200 mL of the mixture with constant stirring at 1200 rpm at 37 °C for 60 min. The solutions were centrifuged at 4000 rpm for 20 min, washed twice, and the particles were freeze-dried on a SCIENTZ-12N Vacuum Lyophilizer (Ningbo Scientz Biotechnology, Shanghai, China) to obtain the nanoparticle powders.

### 2.7. Characterization of Biosynthesized Nanoparticles

#### 2.7.1. UV-Visible Spectrum

The biosynthesis of ZnONPs, TiO_2_NPs, and CsNPs using *S. apiospermum* CF was verified by a UV-Vis spectrophotometer in accordance with the procedure of Javed et al. [32], with some adjustments. Briefly, freeze-dried samples were dissolved separately, and 3 mL of each solution was scanned at wavelengths from 200 to 800 nm using a spectrophotometer (Shimadzu, Kyoto, Japan; resolution: 1 nm). 

#### 2.7.2. Fourier Transform Infrared (FTIR) Measurements

FTIR tests were carried out to investigate the surface features of the nanoparticles and confirm the presence of functional groups, according to the approach of Fouad et al. [33]. Briefly, powder samples of ZnONPs, TiO_2_NPs, and CsNPs biosynthesized using *S. apiospermum* CF were immersed in a KBr matrix. The samples were scanned using a Fourier transform infrared spectrometer (Vectore 22 Bruker, Karlsruhe, Germany) from 400 to 4000 cm^−1^ at a resolution of 4 cm^−1^.

#### 2.7.3. X-ray Diffraction (XRD) Measurements

The XRD technique was utilized to identify the crystalline nature and structure of the ZnONPs, TiO_2_NPs, and CsNPs biosynthesized using *S. apiospermum* CF by an X’pert-Pro diffractometer (Almelo, The Netherlands) with a detector voltage of 45 kV and a current of 20 mA. The diffractogram was captured throughout the 2θ range of 20–90 degrees at a scan rate of 4 min^−1^ [34].

#### 2.7.4. SEM and EDS Measurements

Per the technique of Fouad et al. [33], with minor adjustments, the surface structure, size, and morphological structure of ZnONPs, TiO_2_NPs, and CsNPs biosynthesized using *S. apiospermum* CF were investigated. The samples of biosynthesized ZnONPs, TiO_2_NPs, and CsNPs were dissolved in 100% ethanol and then formed a thin film. After that, the specimens were analyzed by scanning electron microscopy (SEM) (TM1000 Hitachi, Tokyo, Japan). In addition, energy dispersive spectroscopy (EDS) was utilized to determine the elemental composition. For the analysis, a 3 kV acceleration voltage was utilized.

#### 2.7.5. Dynamic Light Scattering (DLS) and Zeta Potential (ZP)

The mean hydrodynamic size, distribution of size (PDI), and ZP measurement of ZnONPs, TiO_2_NPs, and CsNPs biosynthesized using *S. apiospermum* CF were detected using Nano Particle Analyzer Zetasizer S-90 Malvern instruments (Malvern, Worcester, UK).

### 2.8. Termiticidal Activity

The termiticidal activity of 7 concentrations (0, 62.5, 125, 250, 500, 750, and 1000 µg/mL) of ZnONPs, TiO_2_NPs, and CsNPs biosynthesized using *S. apiospermum* CF against workers of *O. formosanus* was assessed using Smith’s techniques [32], with slight modifications. Whatman grade 2 qualitative filter papers were soaked with 0.5 mL of the already mentioned concentrations of ZnONPs, TiO_2_NPs, and CsNPs. One filter paper was placed in each Petri dish after the papers had been allowed to dry for 5 min at room temperature. Filter papers soaked with 0.5 mL of double-distilled water (ddH_2_O) were included as a control. For every dish, ten *O. formosanus* workers were assigned plus pre-ground *O. fragrans* wood after combination with the same treatment. The plates were incubated under a constant condition of ≤27 °C, RH ≤ 60, L/D = 15 h:9 h. After the treatments, the mortality rate was recorded to monitor the termiticidal impact of ZnONPs, TiO_2_NPs, and CsNPs. At 48 h, the LC_50_ and LC_90_ were calculated. 

### 2.9. Morphological and Histological Observations Post-Exposure to Biosynthesized Nanoparticles

For the morphological observations, the methodology described by Neves Filho et al. [35] was followed, with slight modifications. *O. formosanus* workers were exposed to the LC_90_ of ZnONPs, TiO_2_NPs, and CsNPs for 48 h, while the control group was exposed to ddH_2_O. Subsequently, the workers were gently collected, washed twice with ddH_2_O, and then immersed overnight in a 2.5% glutaraldehyde solution. The samples were rinsed with PBS (phosphate-buffered saline) and treated with 2% aqueous OsO_4_ for 1 h. After additional cleaning with PBS, the samples were dehydrated with a series of ethanol grades. Following that, the samples were coated with gold powder using a sputter coater. Finally, they were examined using a scanning electron microscope (TM1000 Hitachi, Japan).

For the histological observations, the technique outlined by Al-Mehmadi et al. [36] was employed, with minor adjustments. *O. formosanus* workers were exposed to both the LC_50_ and LC_90_ of ZnONPs, TiO_2_NPs, and CsNPs for 48 h. The collected workers underwent two gentle washes with ddH_2_O and were then fixed in a 4% paraformaldehyde solution for 48 h. Subsequently, an ascending series of ethanol solutions was used for dehydration, followed by cleaning with a xylene solution. The workers were then embedded in melted paraffin blocks, and 5 μm-thick sections were obtained using a rotary microtome. H and E (hematoxylin and eosin) staining was performed on the sections, which were subsequently analyzed using a light microscope (Leica DM500, Hong Kong, China).

### 2.10. Statistical Analysis

The experiments were performed with three technical replicates. The proportion of mortality was determined using probit analysis, and statistics for LC_50_ and LC_90_ were obtained with 95% confidence intervals. The data were examined using the software SPSS version 21 (SPSS Inc., Chicago, IL, USA). In every data analysis performed to assess the differences between the control and treatment groups, a *p*-value < 0.05 was considered statistically significant.

## 3. Results and Discussion

### 3.1. Isolation and Identification of Rhizosphere Fungi

**Figure 1:**
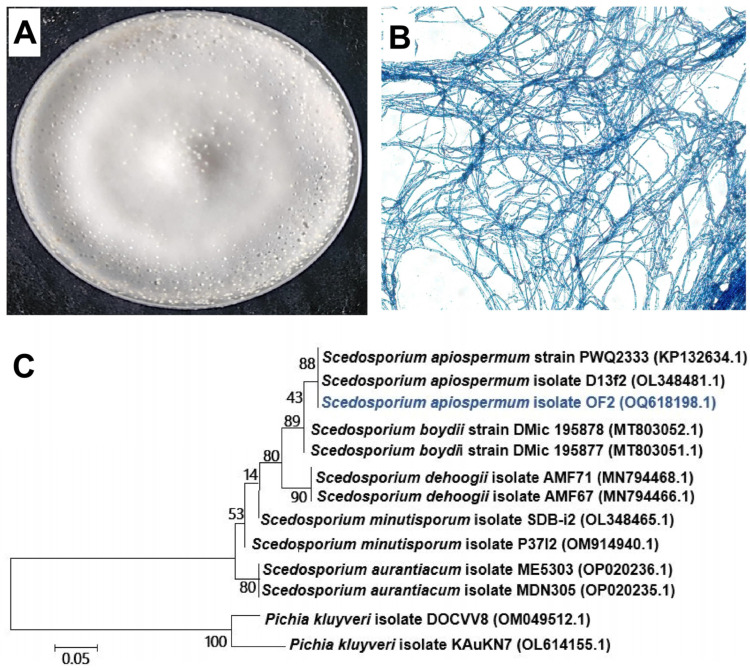
Fungal growth, mycelial development, and phylogenetic tree of the rhizosphere fungus *S. apiospermum* strain OF2 isolated from *O. fragrans*. (**A**) *S. apiospermum* strain OF2 on PDA medium. (**B**) Mycelial growth examined by light microscopy at 40× magnification. (**C**) Phylogenetic tree constructed using ITS rRNA gene sequences. Bootstrap analysis (1000 replicates) Bars: 0.05 substitutions per nucleotide position.

Eight species of fungi were isolated from the rhizosphere of *O. fragrans* due to the *O. formosanus* termite preference for sweet Osmanthus wood [37]. One of them, namely OF2, was selected based on its ability to biosynthesize ZnONPs, TiO_2_NPs, and CsNPs. Using the ITS rRNA, this isolate was examined for molecular identification and DNA sequencing analysis. The result showed that the OF2 ITS rRNA sequence is closely similar to sequences that belong to *Scedosporium* species when it was matched using the BLAST (basic local alignment search tool) from the NCBI (National Centre for Biotechnology Information) website. After that, homologous sequences and the 1ITS rRNA sequences of OF2 were gathered and utilized to build a phylogenetic tree based on genetic distance. OF2 was categorized as *Scedosporium apiospermum*, according to the phylogenetic analysis (Figure 1).

### 3.2. Biosynthesis of Nanoparticles

**Figure 2:**
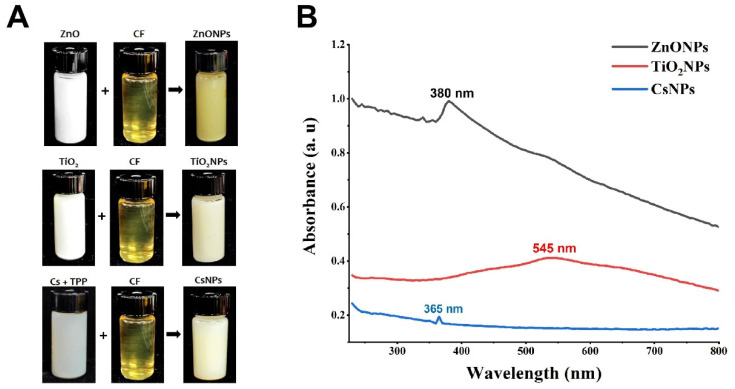
Preparation procedure of ZnONPs, TiO_2_NPs, CsNPs, and UV-Vis spectra. (**A**) Reagents before and after the reaction. (**B**) UV-Vis spectra showing the peaks of ZnONPs, TiO_2_NPs, and CsNPs.

The results of the study demonstrated that when 100 mL of CF from each of the eight isolated fungi was mixed separately with 100 mL of ZnO, TiO_2_, and chitosan, only the CF from the *S. apiospermum* strain was able to induce visible color changes. Specifically, the color changed from white to yellow when the *S. apiospermum* CF was mixed with ZnO, and to a creamy white when mixed with TiO_2_. Furthermore, the color turned yellowish-white when the *S. apiospermum* CF was mixed with chitosan (Figure 2A). Many previous studies have indicated that a color change is a critical initial indicator of the synthesis of nanomaterials, and the specific color variations observed are often attributed to the different materials used in the synthesis process. For example, in accordance with our results, the color of the ZnO solution was altered from colorless to opalescent when mixed with the CF of *Halomonas elongate*, while the color of TiO_2_ turned from white to milky when mixed with the same CF [38], whereas the color of the chitosan solution shifted from pale yellow to white when mixed with the extract of *Pelargonium graveolens* [39]. Visual observation of the biosynthesis of ZnONPs, TiO_2_NPs, and CsNPs using *S. apiospermum* CF was also confirmed by UV spectroscopy. The results showed that curves formed in different locations depending on the element used in the installation. The peak appeared at 380 nm for ZnONPs, 545 nm for TiO_2_NPs, and 365 nm for CsNPs (Figure 2B). In keeping with our study, Ogunyemi et al. [40] revealed a prominent absorption band of ZnONPs in the range of 380–386 nm. Abdallah et al. [41] reported that the greatest peak of CsNPs was measured to be between 320 and 360 nm.

### 3.3. Characterization of Nanoparticles

#### 3.3.1. FTIR

**Figure 3:**
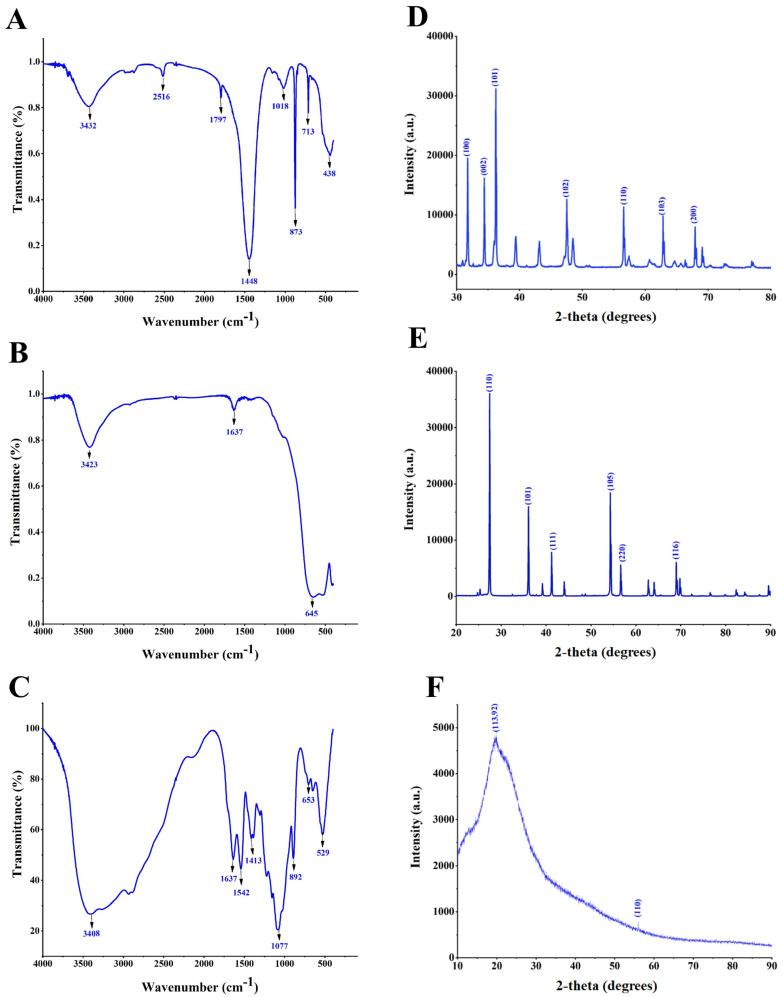
Fourier transform infrared spectra and X-ray diffraction spectra of biosynthesized nanoparticles. (**A**) FTIR spectra of ZnONPs; (**B**) FTIR spectra of TiO_2_NPs; (**C**) FTIR spectra of CsNPs; (**D**) XRD spectra of ZnONPs; (**E**) XRD spectra of TiO_2_NPs; and (**F**) XRD spectra of CsNPs.

FTIR measurements were used to investigate biomolecules for the presence of various functional groups that bond with nanoparticles as a result of reduction and stabilization. To identify the functional groups, the discovered intensity bands were compared to standard values. The responses of the biosynthesized nanoparticles were collected between 500 and 4000 cm^−1^ (Figure 3). The distinctive bands for ZnONPs were discovered at 3432, 2516, 1797, 1448, 1018, 873, 713, and 438 cm^−1^ (Figure 3A). The broad absorption peaks at 3432 cm^−1^ and 2516 cm^−1^ were due to the O-H extending vibrations of water and the free C-H group. The relative absorption peaks at 1448 cm^−1^ for methylene folding vibration could be attributed to the protein content. The bands at 1018 and 873 cm^−1^ were attributed to the amine N-H bending vibration and the primary alcohol C-O stretching group, respectively. The band between 713 and 438 cm^−1^ conformed to ZnO stretching vibrations, confirming the presence of ZnO. Previous studies detected analogous FTIR peaks for ZnONPs [12,42]. Identified bands for TiO_2_NPs were visible at 3423, 1637, and 645 cm^−1^ (Figure 3B). The O-H stretching vibration was attributed to the larger band at 3423 cm^−1^. The peak at 1637 cm^−1^ was attributed to the vibrations of the OH bonds in water molecules that have been surface-adsorbed on the catalyst’s surface [43]. The peak seen at 645 cm^−1^ was related to the TiO_2_ stretching vibration of nanoparticles [44]. CsNPs were found to have absorption bands at 3408, 1637, 1542, 1413, 1077, 892, 653, and 529 cm^−1^ (Figure 3C). 

The O-H stretching group and the amide I group were responsible for the absorption peaks at 3408 and 1637 cm^−1^, respectively. The N-O stretching group of a nitro compound and C-N stretching vibrations (amide III band) were responsible for the peaks seen at 1542 and 1413 cm^−1^, respectively. Additionally, the C-O stretching group of the primary alcohol and the C=C bending group of alkenes were linked to the peaks found at 1077 and 892 cm^−1^, respectively. The vibration of C≡CH was attributed to the peak values of absorption at 653 cm^−1^. NH and C-O out of plane bending were represented by the peak at 529 cm^−1^. This is consistent with our study by El-Naggar et al. [45], which used green synthesis of CsNPs.

#### 3.3.2. XRD

XRD examination was used to evaluate the crystallinity of biosynthesized ZnONPs, TiO_2_NPs, and CsNPs, which can provide highly helpful information regarding the physical and chemical nature of nanoparticles, as shown in Figure 3. The ZnONP spectrum (Figure 3D) showed seven distinct peaks at 2θ = 31.75°, 34.41°, 36.23°, 47.54°, 56.58°, 62.84°, and 67.93°, which could be indexed as (100), (002), (101), (102), (110), (103), and (200) Miller indices. In agreement with our study, Ali et al. [42] observed lattice planes of biosynthesized ZnONPs by *Escherichia coli* at 100, 002, 101, 102, 110, 103, and 112. However, the TiO_2_NPs spectrum (Figure 3E) displayed six major peaks at 2θ = 27.43°, 36.09°, 41.24°, 54.31°, 56.62°, and 69.00°, which could be indexed as (110), (101), (111), (105), (220), and (116) Miller indices. In accordance with our study, TiO_2_NPs were synthesized using *Aspergillus flavus* [46]. Meanwhile, CsNPs showed distinctive crystalline peaks at 2θ = 20.04° (113 counts/s) and 56.00° (110 counts/s), which were slightly shifted to higher diffraction angles (Figure 3F). This implies that the synthesized chitosan nanoparticles possess a crystalline phase. The minor shift in diffraction positions is most likely owing to a shift in the chemical microstructure of the ligands. There are hypotheses that the induced strain can change the nanocrystallite characteristics of the crystal structure. Using the Debye–Scherrer equation, the catalysts’ average crystallite sizes were ascertained based on the XRD results as D = k λ/(βcos⁡θ). The average crystallite size was determined to be around 13.18 nm for ZnONPs, 10.55 nm for TiO_2_NPs, and 1.02 nm for CsNPs.

#### 3.3.3. SEM and EDS Measurements

**Figure 4:**
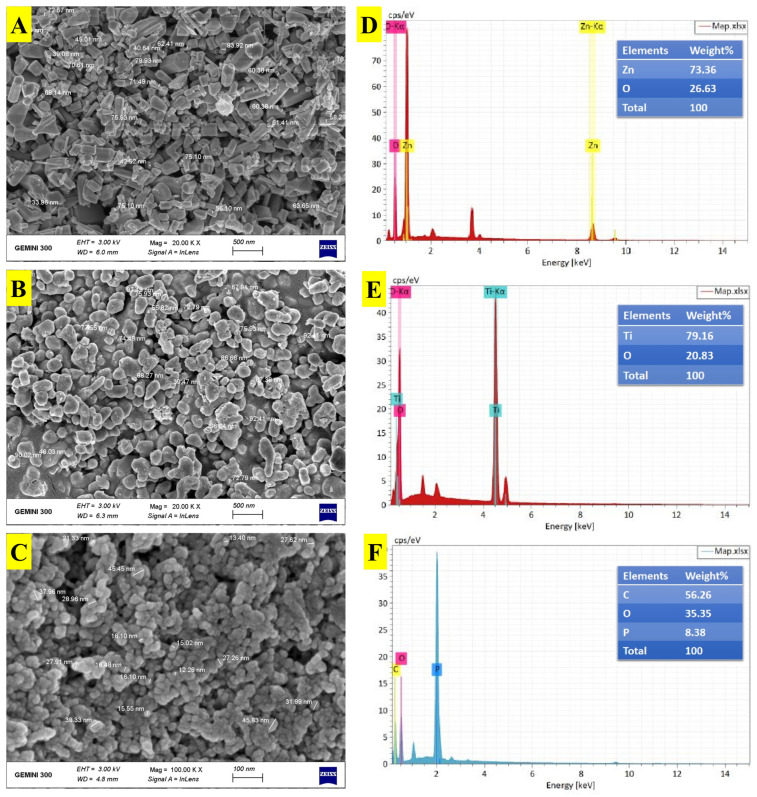
SEM and EDS characterization of biosynthesized nanoparticles. (**A**) SEM images of ZnONPs; (**B**) SEM images of TiO_2_NPs; (**C**) SEM images of CsNPs; (**D**) EDS of ZnONPs; (**E**) EDS of TiO_2_NPs; and (**F**) EDS of CsNPs.

Investigating the surface structure, shape, and size of biosynthesized ZnONPs, TiO_2_NPs, and CsNPs using SEM examination is a valuable technique [47]. According to SEM investigations of ZnONPs, the particles have hexagonal rod morphologies and have a size range of 33.96 to 96.04 nm (Figure 4A). Similar reports have been provided by Fu et al. [48]. The TiO_2_NPs are entirely spherical in shape and have diameters ranging from 51.47 to 97.49 nm (Figure 4B). In line with Al Masoudi et al. [44], biosynthesized TiO_2_NPs have a homogeneous spherical structure and a diameter that falls between 10 and 30 nm. The SEM picture revealed that the biosynthesized CsNPs have a spherical form and a particle size range of 12.28 to 45.83 nm (Figure 4C). According to Thamilarasan et al. [49], CsNPs have a predominately spherical form and range in size from 20 to 100 nm. EDS analysis was used to confirm the elemental composition of the biosynthesized ZnONPs, TiO_2_NPs, and CsNPs. The percentage elemental composition of ZnONPs was revealed by the EDS spectrum to be zinc (73.36%) and oxygen (26.64%) (Figure 4D). Ali et al. [50] concur with our findings. For TiO_2_NPs, titanium (79.16%) and oxygen (20.83%) were recorded (Figure 4E). Murugan et al. [51] confirmed the presence of strong signals for titanium and oxygen atoms in the analyzed samples. The percentage elemental composition of CsNPs was revealed by EDS peaks, which consisted of carbon (56.26%), oxygen (35.35%), and phosphorus (8.38%) (Figure 4F). Sotelo-Boyás et al. [52] noted the same ingredients in various percentages. 

Taran et al. [38] reported that *H. elongata* produced ZnONPs and TiO_2_NPs with average particle diameters of 98 nm and 26 nm, respectively, and PDI values of 0.45 and 0.39, according to our findings. According to El-Naggar et al. [45], CsNPs have a positive charge because of their great stability and increased electrostatic repulsion. In a previous study, it was recorded that ZP values close to zero mean that the particles are flocculating. However, the nanoparticles in this study had ZP values that were far from zero, which means that they are stable [53].

#### 3.3.4. DLS and ZP Measurements

**Figure 5:**
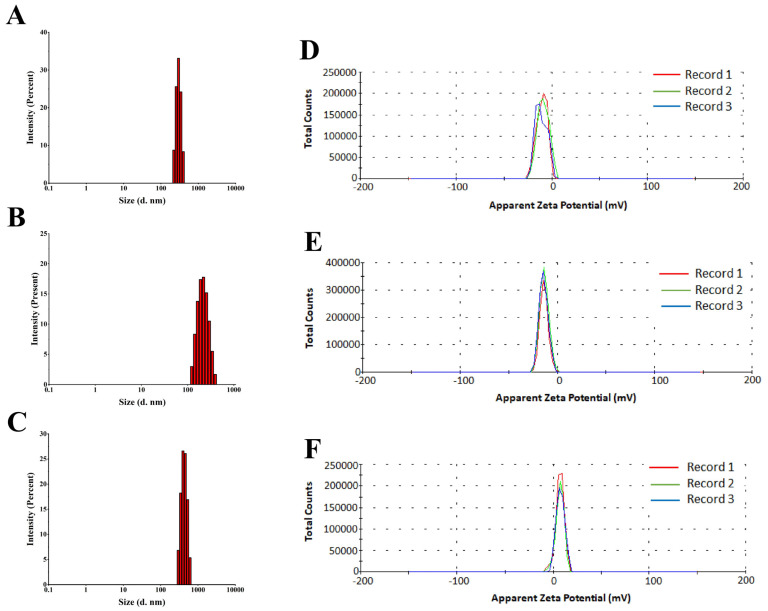
DLS and ZP distribution of biosynthesized nanoparticles. (**A**) DLS distribution of ZnONPs; (**B**) DLS distribution of TiO_2_NPs; (**C**) DLS distribution of CsNPs; (**D**) ZP distribution of ZnONPs; (**E**) ZP distribution of TiO_2_NPs; and (**F**) ZP distribution of CsNPs. Reruns of the experiment are represented by Records 1–3.

According to the DLS study, ZnONPs, TiO_2_NPs, and CsNPs biosynthesized using *S. apiospermum* CF had a median particle size of 295 d. nm, 220 d. nm, and 396 d. nm, with PDI values of 0.371, 0.361, and 0.449, respectively. The ZP of the biosynthesized ZnONPs, TiO_2_NPs, and CsNPs was investigated; nevertheless, it is frequently the only method available to detect the surface charge and stability of NPs. As shown in Figure 5, the ZP value in this study was −10 mV for ZnONPs, −13.7 mV for TiO_2_NPs, and 7.21 mV for CsNPs. Additionally, the ZP distribution has just one peak, which shows superb nanoparticle homogeneity. 

### 3.4. Termiticidal Activity

**Figure 6:**
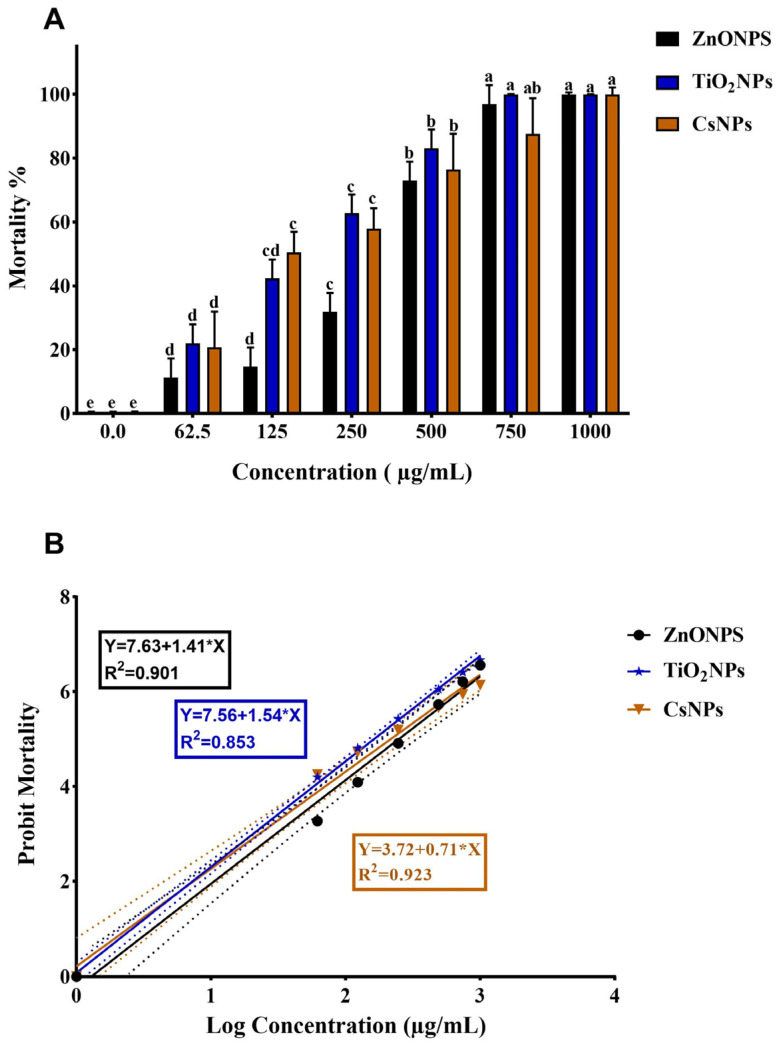
**The** toxic effect of biosynthesized ZnONPs, TiO_2_NPs, and CsNPs on *O. formosanus* workers 48 h after treatment with various concentrations. (**A**) Mortality rate versus concentration. (**B**) Probit regression line of *O. formosanus* exposed to different interval concentrations of biosynthesized nanoparticles. Means ± SD values with distinct letters (a–e) are significantly different at the level of *p* < 0.05 according to Duncan’s test.

ZnONPs, TiO_2_NPs, and CsNPs were evaluated for their termiticidal activity against *O. formosanus* workers at several concentrations (0, 62.5, 125, 250, 500, 750, and 1000 µg/mL). Following the treatments, the death rate started to be observed at 24 h. However, the LC_50_ and LC_90_ were determined at 48 h because this is when the effects of the biosynthesized nanoparticles on termites started to demonstrate significant efficiency, whereas the mortality rate was a negligible percentage at 24 h. Table 1 shows that the biosynthesized ZnONPs, TiO_2_NPs, and C_S_NPs exhibited toxicity toward *O. formosanus* with LC_50_ values of 260.9 µg/mL, 147.5 µg/mL, and 183.5 µg/mL, respectively, after 48 h of treatment, while LC_90_ values were 724.5 µg/mL, 512.9 µg/mL, and 1220.3 µg/mL, respectively, considering the angular coefficient (line slope) (Figure 6). The mortality rate rose with increasing concentration; for ZnONPs, TiO_2_NPs, and CsNPs, the mortality rates were 100%, 100%, and 97.67%, respectively, at the maximum concentration (1000 µg/mL). According to this investigation, it was found that TiO_2_NPs were more toxic to *O. formosanus* compared to the other nanoparticles (Figure 6A).

The use of biosynthesized ZnONPs, TiO_2_NPs, and C_S_NPs against *O. formosanus* has never been documented before. Furthermore, it was observed that the direct application of ZnONPs in soil is deemed safe, addressing potential public concerns regarding food safety associated with nano-enabled agriculture [54]. Studies on nanoparticles’ effectiveness against several other insects abound. For instance, Gutiérrez-Ramírez et al. [55] evaluated the insecticidal effects of ZnONPs and TiO_2_NPs on *Bactericera cockerelli*. Zaki et al. [56] identified the efficacy of TiO_2_NPs as a nanopesticide against *Spodoptera littoralis* larvae by administering castor bean leaves treated with TiO_2_NPs as part of their diet. Asghar et al. [57] investigated the biological synthesis of Ag and ZnO nanoparticles against *Helicoverpa armigera.* Santos et al. [58] documented the insecticidal effects of mycosynthesized AgNPs against *Plutella xylostella* (*P. xylostella*). The study involved feeding cabbage leaves that had been dipped in AgNPs to the insects, demonstrating the nanoparticles’ pesticidal properties. Wu et al. [59] reported the insecticidal effects of mycosynthesized CsNPs against *P. xylostella* by applying them through a spray onto cabbage leaves. Alfy et al. [60] conducted a study investigating the insecticidal properties of CsNPs on *Meloidogyne incognita*, *Locusta migratoria*, and *Spodoptera littoralis*.

### 3.5. Morphological Observations Post-Exposure to Biosynthesized Nanoparticles

**Figure 7:**
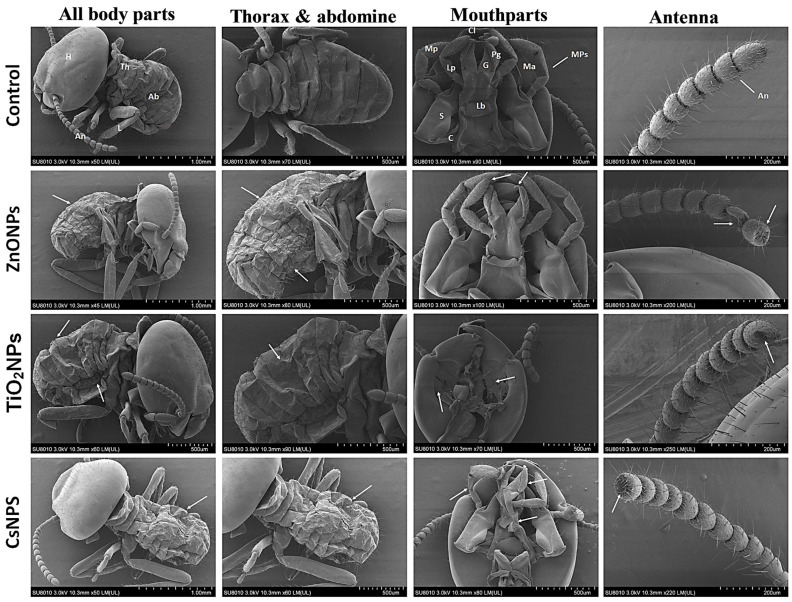
Morphological analysis of *O. formosanus* workers after exposure to ZnONPs, TiO_2_NPs, and CsNPs in comparison to the control. H—head; Th—thorax; Ab—abdomen; An—antennae; Mps—mouthparts; Cl—clypeus; Ma—mandibles; C—cardo; S—stipes; Mp—maxillary palp; Lb—labium; Lp—labial palp; G—glossa; Pg—paraglossa. The arrows point to damage to *O. formosanus* workers.

Figure 7 depicts the morphological structure of *O. formosanus* workers after 48 h of exposure to ZnONPs, TiO_2_NPs, and CsNPs at the LC_90_. In comparison to the control group, the data showed that the treated workers had several deformities. The control group of *O. formosanus* workers had distinct structures of all body parts, including the head with antennae and mouthparts, the thorax with legs, and the abdomen. The most commonly observed deformities of the treated termites were a shrunken cuticle for the tergum, sternum, and pleura; damage to different mouthparts, such as the mandibula, clypeus, labial palp, labium, glossa, paraglossa, cardo, stipes, and maxillary palp; damage at the terminal flagellomeres of the antennae; and leg atrophy. SEM investigations revealed that ZnONPs, TiO_2_NPs, and CsNPs produced significant structural changes in the abdominal cuticle, mouthparts, antennae, thoracic, and leg of *O. formosanus*, particularly in workers exposed to TiO_2_NPs.

The SEM results were not compared to the earlier investigations because there is not much information in the literature regarding how biosynthesized nanoparticles affect *O. formosanus*. The biosynthesized nanoparticle may enter exposed insects through inhalation, contact, or ingestion [61]. Nanoparticles have been reported to be able to pass through the exterior layer of an insect’s skin, spiracles, mouth apertures, anal prologs, setae, and abdominal prolegs [62]. When nanoparticles penetrate the cuticles, they have adverse effects on the physiology and morphology of insects. Through the utilization of triboelectric forces and the surface effect, nanostructured alumina absorbs onto the wax layer, causing the cuticle to become dehydrated [63]. This study’s findings are supported by Nasser et al. [37], who observed modifications in *O. formosanus* workers following treatment with *Myristica fragrans* and *Eucalyptus globulus* nanoemulsions.

### 3.6. Histological Observations Post-Exposure to Biosynthesized Nanoparticles 

**Figure 8:**
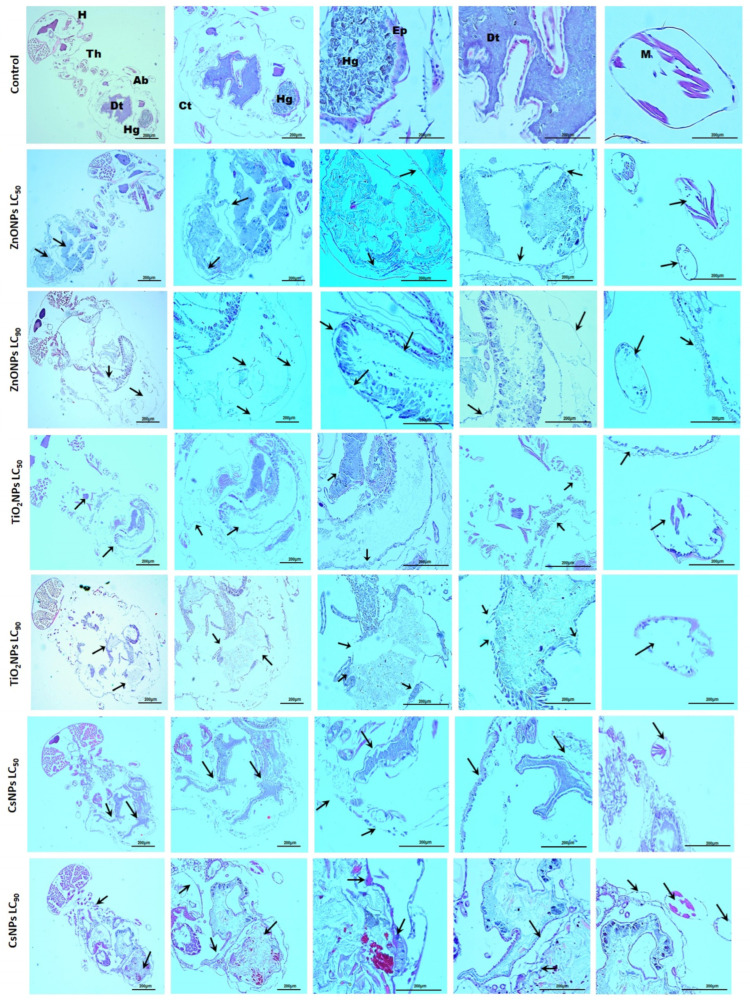
Histological analysis of *O. formosanus* workers following exposure to ZnONPs, TiO_2_NPs, and CsNPs in comparison to the control. H—head; Th—thorax; Ab—abdomen; Ct—cuticle; Dt—digestive tract; M—muscle; Hg—hindgut. The arrows point to damage to *O. formosanus* workers.

The histological studies of *O. formosanus* workers treated with ZnONPs, TiO_2_NPs, and CsNPs for 48 h at the LC_50_ and LC_90_ are shown in Figure 8. In comparison with the control, termites exposed to ZnONPs, TiO_2_NPs, and CsNPs, especially at high concentrations, exhibited significant damage to the normal histological structure. According to these studies, epithelial cells under treatment showed reduced intercellular contacts with neighboring cells, swollen cells, and detachment from the basal lamina, as well as nuclear degradation and muscle degradation, whereas control workers exhibited intact and undamaged entire body regions. There was some leakage of the gut contents. It has been noted that nanomaterials can pass through an insect’s mouth apertures, spiracles, anal prologs, and abdominal prolegs in addition to the outer layer of skin [62]. Insect physiology and morphology are negatively impacted by nanoparticles that break through the cuticles. Nanostructured alumina absorbs onto the wax layer by exploiting the surface effect and triboelectric forces, which dehydrate the cuticle [63]. The midgut is a vital part of the insect that is related to nourishment acquisition and digestion enzyme production. Furthermore, the integrity of the midgut can be studied as an indication of sensitivity to a variety of harmful substances [64]. The outcomes of this investigation are corroborated by Nasser et al. [37], who saw changes in the typical structure of *O. formosanus* workers after treatment with *M. fragrans* and *E. globulus* nanoemulsions. Following treatment with biosynthesized silver nanoparticles, Abou El-Enain et al. [65] showed histopathological indicators in the midgut tissues of *Agrotis ipsilon*.

## 4. Conclusions

According to our knowledge, this is the first time biosynthesized ZnONPs, TiO_2_NPs, and CsNPs nanoparticles have been used to control *O. formosanus*. The biosynthesized ZnONPs, TiO_2_NPs, and CsNPs exhibit high efficacy as termiticidal agents against *O. formosanus* in addition to the morpho-histological changes. Morphological investigations revealed shrunken cuticles, damage to various mouthparts, damage at the terminal flagellomeres of the antennae, and leg atrophy. The histological examination revealed that the epithelial cells had reduced intercellular contacts with neighboring cells, detachment from the basal lamina, nuclear degradation, and muscle degradation. There was some leakage of the gut contents. This investigation highlights the potential of ZnONPs, TiO_2_NPs, and CsNPs as safe alternatives for *O. formosanus* management and recommends further necessary research that will lead to the implementation of the results in practice.

## Figures and Tables

**Table 1:** Toxicity of ZnONPs, TiO_2_NPs, and CsNPs against workers of *O. formosanus* 48 h after treatment.

Treatment	No.	Concentrations (µg/mL)	Mortality (%) ± SD	LC_50_ (95% LCL-UCL)	LC_90_ (95% LCL-UCL)	X^2^
ZnONPs	1	0.00	0.00 ± 0.00 ^e^	260.90 (179.90–366.41)	724.50 (491.00–1505.81)	3.05
	2	62.5	11.30 ± 5.93 ^d^			
	3	125	14.73 ± 5.93 ^d^			
	4	250	31.86 ± 5.93 ^c^			
	5	500	72.97 ± 5.93 ^b^			
	6	750	96.95 ± 5.93 ^a^			
	7	1000	100.00 ± 0.00 ^a^			
TiO_2_NPs	1	0.00	0.00 ± 0.00 ^e^	147.50 (84.60–217.90)	512.92 (328.91–1279.10)	1.53
	2	62.5	22.04 ± 5.87 ^d^			
	3	125	42.38 ± 5.87 ^cd^			
	4	250	62.73 ± 5.87 ^c^			
	5	500	83.07 ± 5.87 ^b^			
	6	750	100.00 ± 0.00 ^a^			
	7	1000	100.00 ± 0.00 ^a^			
CsNPs	1	0.00	0.00 ± 0.00 ^e^	183.51 (78.23–308.90)	1220.30 (614.90–8865.2)	1.11
	2	62.5	20.29 ± 10.86 ^d^			
	3	125	52.89 ± 10.86 ^c^			
	4	250	56.52 ± 6.27 ^c^			
	5	500	74.63 ± 10.86 ^b^			
	6	750	85.51 ± 10.86 ^ab^			
	7	1000	97.67 ± 2.23 ^a^			

LC_50_—lethal concentration that kills 50% of insects, LC_90_—lethal concentration that kills 90% of insects, LCL—lower confidence limit, UCL—upper confidence limit, SD—standard deviation, ^a–e^ letters refer to significant differences based on Duncan’s test at *p* ≥ 0.05 between the control and other treatments, X^2^—Chi square.

## Data Availability

The data are contained within the manuscript.
